# Telocytes in regenerative medicine

**DOI:** 10.1111/jcmm.12594

**Published:** 2015-06-08

**Authors:** Yihua Bei, Fei Wang, Changqing Yang, Junjie Xiao

**Affiliations:** a Regeneration and Ageing Lab, Experimental Center of Life Sciences, School of Life Science, Shanghai UniversityShanghai, China; b Division of Gastroenterology and Hepatology, Digestive Disease Institute, Shanghai Tongji Hospital, Tongji University School of MedicineShanghai, China

**Keywords:** telocytes, regenerative medicine, regeneration, stem cells, progenitor cells, interstitial cells

## Abstract

Telocytes (TCs) are a distinct type of interstitial cells characterized by a small cell body and extremely long and thin telopodes (Tps). The presence of TCs has been documented in many tissues and organs (go to http://www.telocytes.com). Functionally, TCs form a three-dimensional (3D) interstitial network by homocellular and heterocellular communication and are involved in the maintenance of tissue homeostasis. As important interstitial cells to guide or nurse putative stem and progenitor cells in stem cell niches in a spectrum of tissues and organs, TCs contribute to tissue repair and regeneration. This review focuses on the latest progresses regarding TCs in the repair and regeneration of different tissues and organs, including heart, lung, skeletal muscle, skin, meninges and choroid plexus, eye, liver, uterus and urinary system. By targeting TCs alone or in tandem with stem cells, we might promote regeneration and prevent the evolution to irreversible tissue damage. Exploring pharmacological or non-pharmacological methods to enhance the growth of TCs would be a novel therapeutic strategy besides exogenous transplantation for many diseased disorders.

- History of telocyte identification
- Electron microscopic features of TCs
- TC immunohistochemistry
- Distribution of TCs
- Cellular junctions of TCs and their functional implications
- TCs in tissue repair/regeneration
  Heart
  Lungs and trachea
  Skeletal muscle
  Skin
  Meninges and choroid plexus
  Eye
  Liver
  Uterus
  Urinary system
- Targeting TCs as a potential therapeutic strategy
- Summary

## History of telocyte identification

Telocytes (TCs), a novel type of interstitial cells, was firstly described in 2010 by Popescu’s group as a case of serendipity 1. Although TCs have been described between 2005 and 2009 by Popescu’s group as interstitial Cajal-like cells (ICLC), they are completely distinct from ‘interstitial cells of Cajal’ (ICC) in ultrastructures, immunochemical features, and gene and protein profiles 1–7. To avoid further confusion with ICC, Popescu’s group renamed ICLC with ‘TELOCYTE’ by using the Greek affix ‘telos’ according to the unique feature of TCs that clearly distinguishes them from all other interstitial cell types: a small cell body and extremely long and thin prolongations termed ‘TELOPODES’ 1.

## Electron microscopic features of TCs

**Figure 1:**
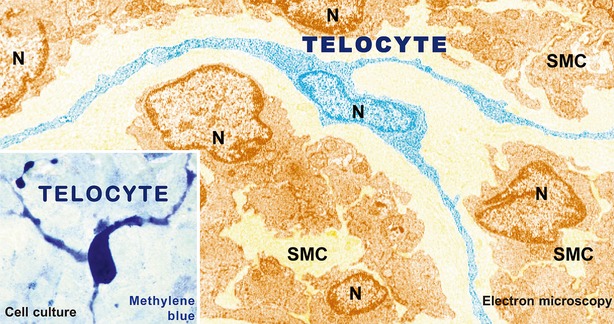
Representative electron microscopy image of a telocyte. A telocyte (TC) with at least three prolongations with several ‘beads’ along telopodes (Tps) is digitally coloured in blue. SMC: smooth muscle cell; N: Nuclei. Original magnification ×6800. Reproduced with permission from 8.

**Figure 2:**
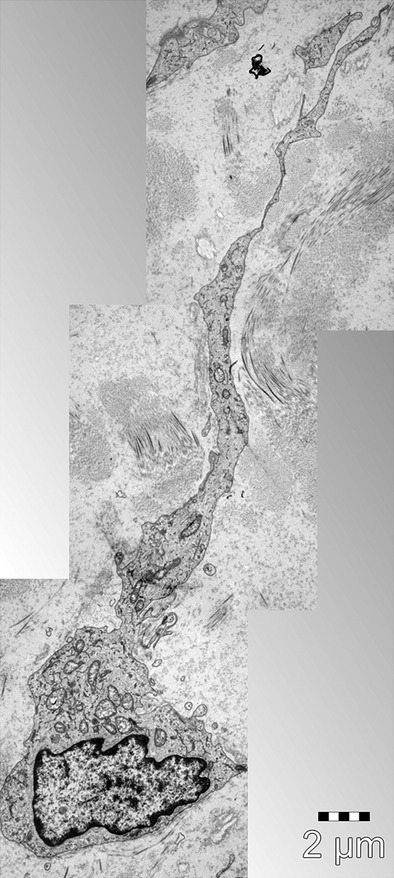
Three sequenced electron microscopy images of a telocyte. A telocyte (TC) with typical long and thin telopode (Tp) extending from the cell body; scale bars: 2 μm. Courtesy of Dr. LM. Popescu, Department of Ultrastructural Pathology, Victor Babeş National Institute of Pathology, Bucharest, Romania.

**Figure 3:**
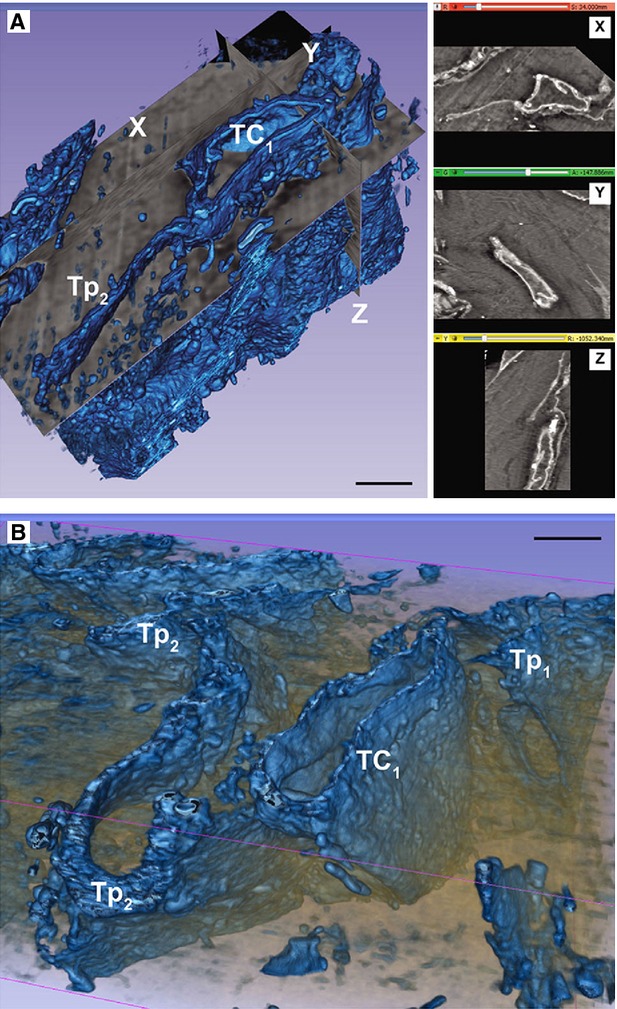
Three-dimensional (3D) reconstruction of a cardiac telocyte. (A and B) automated segmentation of a stack containing a telocyte (TC) with a long (20 ml), narrow (0.2-1 ml) and flat telepode (referred to Tp2 here). The right side of A shows X-Y-Z slice projections; scale bars: 2 ml. Reproduced with permission from 9.

Cells with telopodes (Tps) is the shortest definition of TCs 1. To date, the golden standard for the identification of TCs is transmission electron microscopy 8 (Fig.1). The cell body of TCs has a nucleus which occupies about 25% of the cell volume, surrounded by a small quantity of cytoplasm containing mitochondria, small Golgi complex, rough and smooth endoplasmic reticulum and cytoskeletal elements. The shape of TCs could be piriform, spindle or triangular, depending on the number of their prolongations ‘Tps’. Tps (normally 1 to 5 for one cell body) are extremely long (tens to hundreds of μm measured by electron microscopy) and thin (below 0.2 μm under resolving power of light microscopy), with moniliform aspect (alternation of thin segments termed podomers and small dilations termed podoms accommodating mitochondria, endoplasmic reticulum and caveolae) 1. Because of the typical cellular prolongations of TCs, sequenced concatenation of electron microscopy images are preferable to better reflect the ultrastructural feature of TCs and Tps (Fig.2). Interestingly, three-dimensional (3D) imaging of human cardiac TCs has recently been shown by focused ion beam-scanning electron microscopy (FIB-SEM) and 3D reconstruction of a human cardiac TC indicated a ribbon-like conformation for Tps and podoms were observed to be bulged from the podomer plane 9 (Fig.3). Of note, the FIB-SEM is currently the most accurate, new and powerful technique used in describing TCs 9.

## TC immunohistochemistry

Although TCs are positive to certain tested markers such as c-kit/CD117, vimentin, CD34 and VEGF, the positivity to these markers varies among different organs and tissues 10 (Table1). In human myometrium, some TCs are c-kit positive 11 and some only CD34 positive but c-kit negative 12, while some of the c-kit positive TCs express also CD34 13. In human urinary tract, TCs are vimentin positive, but CD34, c-kit and VEGF negative 14. Meanwhile, TCs have region-dependent immunohistochemical profiles in the urinary tract according to their different positivity to caveolin-1, oestrogen receptor (ER) and progesterone receptor (PR), which indicates that each region might contain its own subpopulation of TCs 14. Telocytes share some common markers with other types of cells, *e.g*. endothelial cells, which express CD34 15, fibroblasts, positive to vimentin 16 and stem cells, known for c-kit 17, thus it is important to discriminate between TCs and these cells in tissues and cell cultures. For example, primary culture of cardiac TCs and fibroblasts demonstrated that TCs had different immunohistochemical features from fibroblasts. Cardiac TCs were positive for CD34 and c-kit, CD34 and vimentin, and CD34 and PDGFR-β, while fibroblasts were only positive for vimentin and PDGFR-β 18. In addition, TCs have been found different from pericytes in both ultrastructures and immunophenotypes 18–20. Cardiac TCs in primary culture were CD34 positive and α-SMA weak positive while pericytes were CD34 negative but α-SMA positive 18. Besides, the positivity of TCs to PDGFR-α in the human gastrointestinal tract should not be overlooked 21. More recently, it has been reported that podoplanin (D2-40) could be a complementary effective TC immunohistochemical marker in urinary bladder 22. Actually, not a single specific immunomarker allows unequivocal identification of TCs. However, it is currently considered that the double positive labelling for CD34/PDGFR-α and CD34/vimentin (for Tps) is appropriate for the identification of TCs 7. Therefore, immunolabelling, especially double-immunolabelling, remains a useful tool for discrimination between TCs and other cells, as well as for semi-quantitative data analysis.

**Table 1:** TC immunohistochemistry in different organs and tissues

Tissues and organs	TC markers	References
Heart	c-kit, CD34, vimentin, PDGFR-β	15,18,24,26,60,101,102
Lungs and trachea	c-kit, CD34, vimentin, PDGFR-β, Sca-1, VEGF	31,32,107
Skeletal muscle	c-kit, vimentin, PDGFR-β, VEGF, caveolin-1	19,34,35
Skin	c-kit, CD34, vimentin	54,74,75
Meninges and choroid plexus	c-kit	43
Gastrointestinal system	c-kit, CD34, vimentin, PDGFR-α, PDGFR-β	21,57,78
Uterus	c-kit, CD34, vimentin, PDGFR-α, connexin43	11–13,50,52
Urinary system	c-kit, CD34, vimentin, caveolin-1, podoplanin (D2-40)	14,22,42,82

## Distribution of TCs

Since their identification in 2010, TCs have been found existing in fish, reptiles, mammals and humans, and have been identified in the interstitial compartment of many tissues and organs, including heart (myocardium 23,24, epicardium 15, endocardium 25, heart valves 26), vasculature 27–29, pleura 30, lungs and trachea 31–33, placenta 16, skeletal muscle 19,34,35, oesophagus 36,37, intestine 38–40, urinary system 14,41,42, nervous system 43–45, pancreas 46,47, parotid glands 48, prostate 49, uterus 11–13,50–53, skin 54,55, eye 56, liver 57 and bone marrow 58. Also, TCs have been identified among smooth muscle fibres and in the lamina propria of different structures and organs 11–14,38,39,41.

## Cellular junctions of TCs and their functional implications

**Figure 4:**
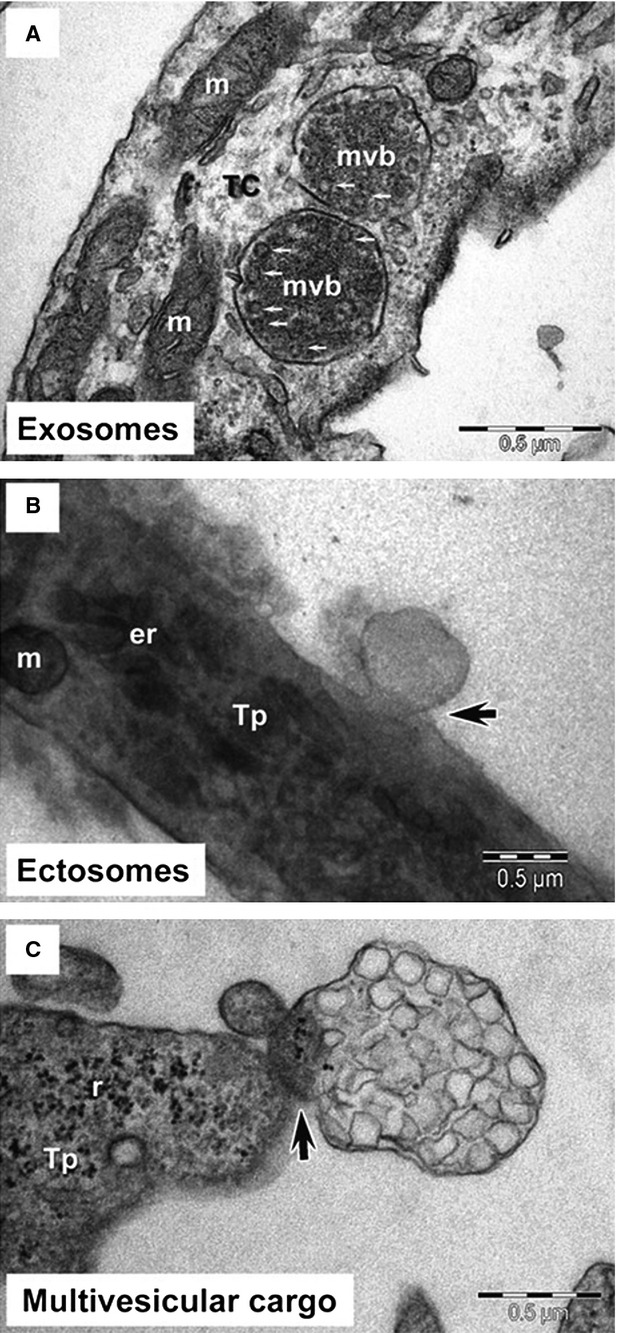
Telocytes secrete extracellular vesicles. Telocytes (TCs) secrete exosomes (A), ectosomes (B), and multivesicular cargo (C). Tp: telopode; mvb: multivesicular bodies; m: mitochondria; er: endoplasmic reticulum; r: ribosome; scale bars: 0.5 μm. Reproduced with permission from 61.

Multiple electron microscopy techniques, including transmission electron microscopy, electron tomography, SEM, FIB-SEM, have demonstrated that TCs have their ‘strategic’ locations in tissues 1. Telocytes can be connected with each other *via* homocellular junctions through their Tps, or be in close vicinity of blood vessels, nerve endings and many other cells (for example in heart, including cardiomyocytes, stem cells, fibroblasts and immunoreactive cells) *via* heterocellular junctions 59. By homo- and heterocellular junctions, TCs can form an interstitial 3D network. Therefore, TCs are critically involved in intercellular signalling *via* paracrine and/or juxtacrine secretion of small molecules, or *via* shed extracellular vesicles which send important macromolecules (*e.g*. RNAs, proteins or microRNAs) to neighbouring cells in normal and diseased states 30,60,61. Three types of extracellular vesicles have been identified in the proximity of cardiac TCs in culture using electron microscopy, including exosomes (45 ± 8 nm in diameter), ectosomes (128 ± 28 nm in diameter) and multivesicular cargos (1 ± 0.4 μm in diameter) 61 (Fig.4). A case in point is that when TCs are transfected with microRNA-21 mimics labelled with Cy5, the recipient cells will become Cy5 positive after incubation with the conditioned medium from TCs, indicating that vesicles are released with microRNAs from TCs 62. Moreover, intestinal TCs display a phagocytic-like property termed endocytosis, which enables themselves to uptake small particles 63. By these properties, TCs may be able to affect or control the activity of the surrounding cells.

Increasing evidence indicated that TCs might be implicated in tissue homeostasis and development 24,64,65, as well as in the pathogenesis of some disorders 66–75. Besides, owing to their close relationship with stem cells and/or progenitor cells in multiple tissues such as heart, lung, skeletal muscle, skin, meninges and choroid plexus, eye and liver, TCs are also supposed to potentially contribute to tissue repair/regeneration 32,34,35,43,54,56,59,75–78. A deeper understanding of how TCs communicate with other cells and take effect in signalling pathway during tissue repair/regeneration will be useful to identify novel therapeutic strategies in regenerative medicine.

## TCs in tissue repair/regeneration

Although the tempting idea of using stem cells and progenitor cells with regenerative potential has been hailed for many years as a promising cell-based therapeutic strategy for tissue repair/regeneration, its actual therapeutic activity is far from satisfactory 79. One of the most important challenges is to establish a well-organized microenvironment which ensures the survival, distribution and regenerative potential of the injected stem cells 80. Stem cell niches are small areas housing stem cells which contain other constructive elements, including interstitial supporting cells, extracellular matrix proteins, blood vessels and neural inputs 81. Growing evidence has indicated that TCs could be located in stem cell niches in a spectrum of organs and tissues, and form a complex interstitial network with resident stem cells, blood vessels, nerve endings and other interstitial components, thus might importantly contribute to tissue repair and regeneration 32,34,35,43,52,54,56,59,75–78,82. The following content of this review will focus on the latest progresses of the potential involvement of TCs in tissue repair and regeneration in heart, lung, skeletal muscle, skin, meninges and choroid plexus, eye, liver, uterus and urinary system.

### Heart

**Figure 5:**
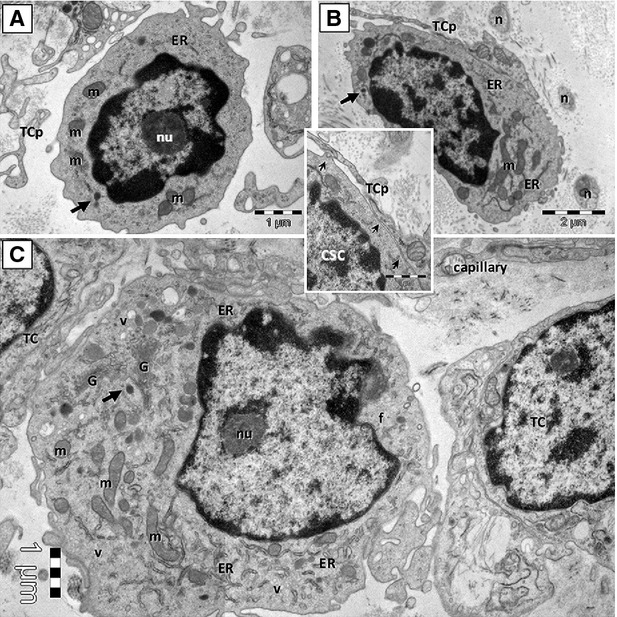
Cardiac telocyte processes are in close contact with cardiac stem cells (CSC). Putative CSC in mouse CSC niche are shown in A and B. Committed cell (an intermediate stage between CSC and cardiomyocyte progenitors, CMP) is shown in C. TCp: telocyte processes; nu: nucleolus; ER: endoplasmic reticulum cisternae; m: mitochondria; n: nerve fibres; G: Golgi apparatus; v: vesicles; f: filaments. Reproduced with permission from 76.

Cardiac stem cells (CSC) and cardiomyocyte progenitors (CMP) are appropriate sources for regenerative medicine strategies for cellular cardiomyogenesis and neovascularization 79,83–88. By electron microscopy, CSC, CMP, as well as the cells with intermediate features between CSC and CMP were identified in epicardial stem cell niche 76 (Fig.5). All these cells with different ultrastructural features were supposed to represent different stages of development and maturation of a unique type of resident stem cell 76. Noteworthy, TCs were also identified in epicardial stem cell niche, in close contact with these resident cardiomyocyte precursors, as well as blood capillaries, nerve endings and other interstitial cells, thus might contribute to make a supportive interstitial network for CSC and CMP to sustain a continuous cardiac renewal process 76. In addition, TCs also existed in human heart valves and could form junctions with resident putative stem (progenitor) cells 26. Telocytes are regarded as important interstitial cells to ‘nurse’ or ‘guide’ putative cardiomyocyte precursors to differentiate and integrate into heart architecture 76,89. Recently, it has been reported that TCs and CSCs represent a small fraction of human cardiac interstitial cells in the range 0.5–1% and 0.1–0.5%, respectively. Furthermore, the number of cardiac TCs and CSCs is decreased in adults *versus* newborns, which might be responsible for reduced cardiac regenerative capacity with ageing process 90. Epicardium is considered as a novel source of cardiac progenitor cells 91. Given that cardiac TCs and epicardium-derived progenitor cells (EDPC) express some common surface molecules such as c-kit and PDGFR-β 15,26,60,92, it should also take into account that TCs might be a subpopulation of EDPC, which could be critically involved in heart development and cardiac regeneration 64.

Cardiac TCs in primary culture is a useful tool to investigate cell functions, including their roles in cardiac regeneration 18,93. Cardiac TCs have stronger adherence and spreading ability of Tps when seeded on fibronectin, and higher dynamics of Tp extension on collagen I, indicating that different matrix proteins might impact TC behaviour in tissue regeneration 93–96. The junctions between TCs and cardiomyocytes are formed by small point contacts with electrondense nanocontacts 97. The junctions between TCs and putative cardiac stem or progenitor cells resemble either stromal synapse or adhaerens junctions 59. In addition, TCs could also form point and/or planar contacts with endothelial cells, pericytes, Schwann cells, as well as other interstitial cells (fibroblasts, mast cells or macrophages) in the heart 59. Thus, TCs are critically involved in the integration of all these heterocellular communications which may be essential for the proliferation, differentiation and maturation of myocardial precursors into new cardiomyocytes 59.

Given that the potential of cardiac regeneration/repair significantly affects the consequences of myocardial infarction (MI), stem cell therapy was recognized as a prompting strategy for ischaemic cardiomyopathy 98,99. The important involvement of TCs in neo-angiogenesis after experimental acute MI has previously been documented 100. The number of TCs was markedly increased in the border zone of MI during the neo-angiogenesis phase after MI, and ultrastructurally, TCs have close connections with endothelial cells of preexisting and neo-formed capillaries 100. It has been supposed that TCs might contribute to neo-angiogenesis *via* paracrine secretion as shown by their positivity to VEGF and NOS2 immunohistochemistry staining 100. Last but not least, several angiogenic microRNAs (let-7e, 10a, 21, 27b, 100, 126-3p, 130a, 143, 155 and 503) are expressed by isolated cardiac TCs 100. In addition, decreased number of cardiac TCs during MI has been demonstrated, while simultaneous transplantation of cardiac TCs in the infarcted and border zones of the heart has been proved effective to reduce the infarction size and improve myocardial function 14 days after MI in rats 101. These beneficial effects of TC transplantation were maintained for at least 14 weeks after MI, probably related to enhanced angiogenesis and decreased myocardial fibrosis, indicating that rebuilding cardiac TC network through transplantation might have both acute and midterm beneficial effects to promote cardiac repair and regeneration following MI 102. More recently, it has been reported that myocardial transplantation of human induced pluripotent stem cell-derived mesenchymal stem cells reduced MI with a presence of interconnecting TCs in the interstitial space of infarcted zone of the heart 103. However, more functional studies *in vivo* and the use of cardiac tissue engineering *in vitro* will help further clarify the functional roles of TCs in cardiac repair and regeneration 60,104–106.

### Lungs and trachea

**Figure 6:**
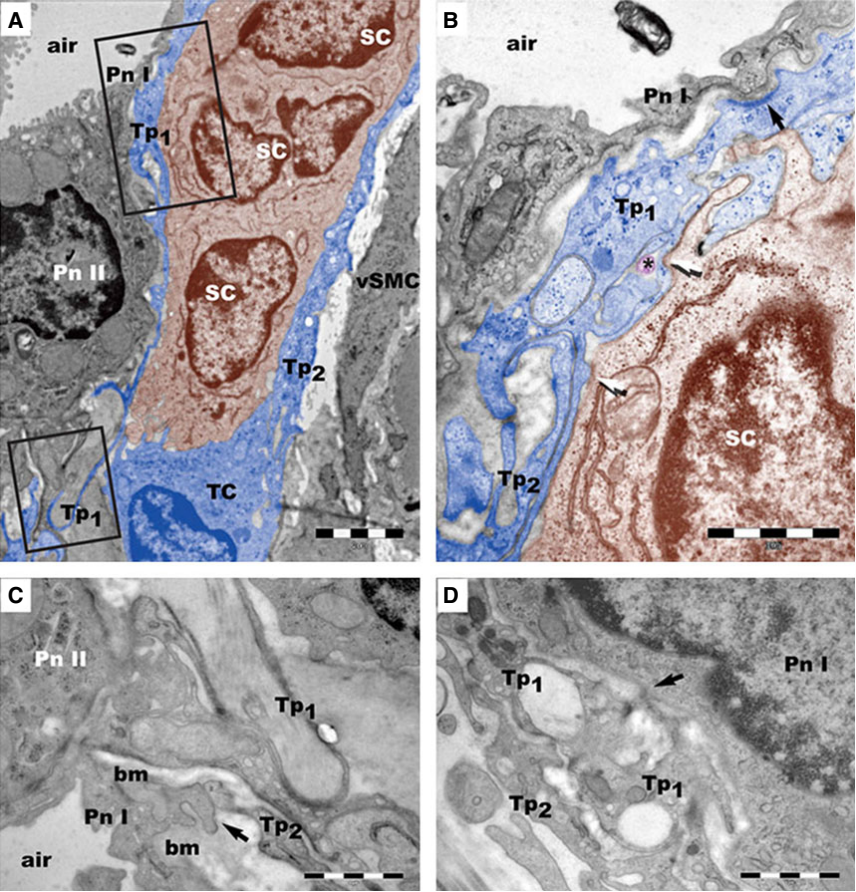
Lung telocytes in stem cell niche. (A) Lung telocyte (TC) is in stem cell (SC) niche. Pn I: type I pneumocytes; Pn II: type II pneumocytes; vSMC: vascular smooth muscle cells. (B) One telopode (Tp1) is shown in contact with a Pn I and a SC. (C) One telopode (Tp2) is shown in contact with a Pn I. (D) A contact point (*arrow*) is found between a telopode (Tp1) and a Pn I. Scale bars: 2 μm (A); 1 μm (B–D). Reproduced with permission from 32.

Telocytes contribute to form a 3D interstitial network in pleura, trachea and lungs, through close associations with fibroblasts, smooth muscle cells, endothelial cells, immunoreactive cells and nerve endings, which suggests the conventional roles of TCs in mechanical support, immune surveillance and intercellular communication and signalling 30,31,33. In pleura, TCs have been found in connection with mononuclear cells, macrophages and mast cells beneath the mesothelium, indicating that TCs might play important roles in mesothelial-cell-induced mesothelial repair 30. In addition, the close connection or junction between TCs and clusters of putative stem cells have been identified in the peribronchiolar spaces or underneath the alveolar epithelial cells in lungs 32 (Fig.6). Telocytes might be potentially involved in the mechanical support for lung stem cell niches, and contribute to the nurse, communication and stimulation of putative stem/progenitor cells in the repair of lung injury, probably through nanocontacts and shed vesicles and/or exosomes 32.

Based on the important fact that TCs connect with multiple cells in the lungs and coordinate the intercellular communications, the potential significance of TCs in the pathogenesis of certain lung diseases has been has been suggested, including pulmonary infectious diseases, chronic obstructive pulmonary disease and interstitial lung disease 67,70. In addition, the gene profiles and proteome features of TCs have been identified in the lungs, especially in comparison with mesenchymal stem cells and fibroblasts 3–6. Multiple genes with regulatory effects in tissue remodelling/repair and vascular basement membrane stability are remarkably up-regulated in lung TCs as compared to fibroblasts, including connective tissue growth factor, Transgelin, Nidogen 1, tissue inhibitor of metalloproteinase 3, collagen type IV, matrix metallopeptidase 3 (Mmp3), Mmp10 and retinol-binding protein 1 4. According to proteomic analysis, myosin-14 and periplakin expressions are up-regulated in lung TCs compared with fibroblasts, suggesting the possible roles of TCs in mechanical sensing, mechanochemical conversion task and tissue remodelling/renewal 3. Besides, several proteins that are highly expressed in extracellular vesicles have been found up-regulated in lung TCs, indicating that TCs might contribute to intercellular signalling and influence stem cell niche fate 3. In addition, comparative gene expressions of chromosome 1-3 have been determined between lung TCs and other cells, such as mesenchymal stem cells, fibroblasts, epithelial cells and lymphocytes 5,6. Noteworthy, the up-regulated expressions of Capn2, Fhl2 and Qsox1 in lung TCs highly supported previous hypothesis that TCs might be implicated in tissue homeostasis and protect against tissue inflammation and fibrogenesis in lung diseases 5. Moreover, it has been demonstrated that lung TCs comprise octamer binding transcription factor 4 (Oct4)-positive cells 107. Since Oct4 is a pluripotency marker expressed in embryonic stem cells responsible for their undifferentiated state, TCs might represent a population of stem cells that potentially contribute to lung regeneration 107. More recently, comparison of protein profiling between human lung TCs and microvascular endothelial cells has shown that TCs are completely different from endothelial cells 2. Meanwhile, bioinformatics analysis has demonstrated the potential involvement of TCs in intercellular communication, oxidative stress, cellular ageing and pro-proliferative effects through the inhibition of apoptosis 2. Further investigations for gene-, protein- and microRNA-expression profiles of TCs will be useful to differentiate TCs from other cell types, as well as to reveal their potential biological roles in lung diseases.

### Skeletal muscle

Adult skeletal muscle has a remarkable regenerative capacity after injury. The activation of the muscle stem cells, also called satellite cells, is a limiting step in muscle regeneration, which enables satellite cells to proliferate, migrate and differentiate into new skeletal myocytes 108. Telocytes have been identified in skeletal muscle interstitium in close vicinity of blood capillaries, nerve fibres, satellite cells and myocytes, suggesting their potential roles in muscle regeneration 34,35. Electron microscopy showed that Tps were extended to neighbouring cells and interconnected by different kinds of junctions, which suggest that TCs potentially contribute to form a 3D interstitial network in skeletal muscle tissue. Shed vesicles/exosomes released by Tps were also detected as previously found in other tissues 34.

**Figure 7:**
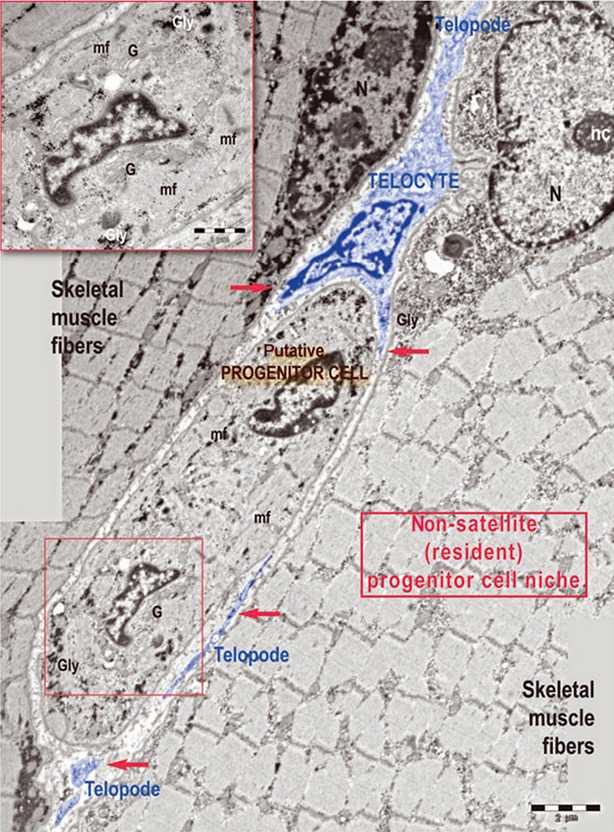
A telocyte (TC) with its telopodes (Tps) around a progenitor cell in skeletal muscle. Tps are indicated with red arrows. mf: myofilaments; Gly: glycogen deposits; G: Golgi complex; N: nucleus; nc: nucleolus. Reproduced with permission from 34.

In addition to muscle satellite cells, non-satellite cells as well as many other types of cells such as bone marrow-derived cells and pericytes contribute consistently to the extraordinary regenerative ability of skeletal muscle 109–112. Of note, TCs have been found in both satellite cell niche and non-satellite progenitor cell niche, forming close contacts with these two types of muscle stem cells 34 (Fig.7). In addition, TCs showed a peculiar migration capacity and formed a cellular network from muscle explants *in vitro*, which might be critical for scaffold guidance of stem/progenitor cells in muscle regeneration 34. Given that TCs in skeletal muscle interstitium are VEGF and PDGFR-β immunopositive both *in situ* and *in vitro*, TCs could also be involved in angiogenesis and vascular stability during tissue repair in skeletal muscle 19,35.

### Skin

**Figure 8:**
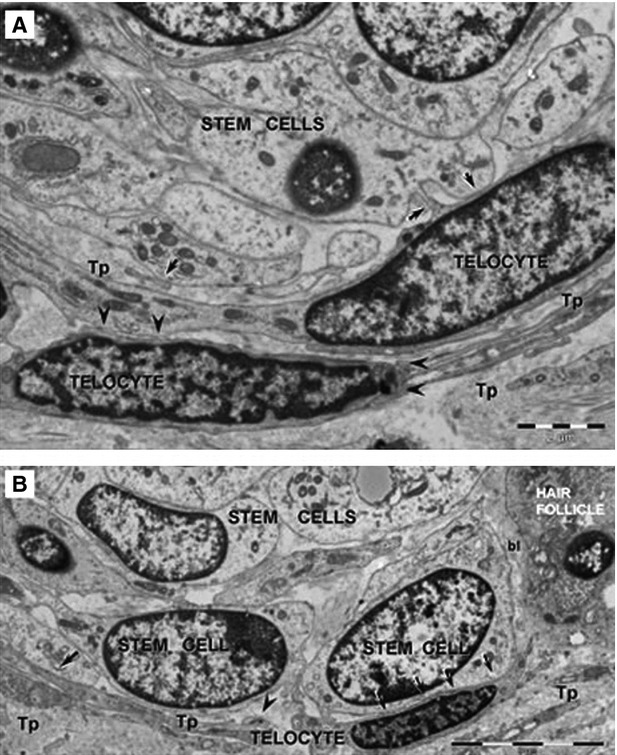
Telocytes bordering stem cells in dermal connective tissue. Telocytes (TCs) formed point contacts (arrows in A) and planar contacts (arrows in B) with stem cells in skin. bl: basal lamina; Tp: Telopode. Reproduced with permission from 54.

The extraordinary regenerative capacity of skin is particularly important for skin repair and regeneration following injury and disease. The presence of TCs in skin has previously been documented. By transmission electronic microcopy, TCs were identified scarce in papillary dermis but numerous in reticular dermis 54. Telocytes are usually lining collagen fibres and elastic fibres, and in close vicinity of mast cells, fibroblasts, adipocytes, blood vessels, nerves and adnexal structures of skin, working as supporting cells to ensure the maintenance of skin homeostasis 54,113. With FIB-SEM technology, extracellular vesicles (approximately *n* = 30 for one TC, 438.6 ± 149.1 nm in diameter) have recently been identified in close vicinity of human dermal TCs 55. Multiple skin stem/progenitor cell compartments have been found throughout all layers of skin, such as hair follicle bulge, interfollicular epidermis, dermal papillae and perivascular space 114,115. It has been demonstrated that TCs were present around the cluster of stem cells in the bulge regions of hair follicles, suggesting that TCs might be implicated in skin repair and regeneration 54 (Fig.8). Since point contacts and planar contacts have been found between TCs and bulge stem cells, it will be of great interest to investigate the potential roles of TCs in activating resident stem cells to proliferate and differentiate during skin regeneration 54.

Systemic sclerosis (SSc), a heterogenous chronic connective tissue disease, is characterized by endothelial dysfunction, immune system disorders, and progressive fibrosis in skin and visceral organs including heart, lung, kidney and oesophagus 116. Interestingly, a progressive reduction and loss, as well as ultrastructural damages of TCs were found in the skin of patients affected by SSc, with relevant differences according to disease subsets (limited/diffuse cutaneous SSc) and stages (early/advance) 75. These phenomena might be related to chronic ischaemic microenvironment of SSc skin, excessive damage and fibrosis of extracellular matrix, and abnormal activation of immune system 75. As TCs contribute to form an organized 3D interstitial network in skin, the reduction and ultrastructural alteration of TCs might be implicated in the imbalance of skin tissue homeostasis. In addition, reduced number of TCs in skin interstitium might be responsible for the loss of control of fibroblast/myofibroblast activity. Last but not least, TCs were progressively reduced around skin stem cell niches in SSc skin, and furthermore, hardly no vascular wall-resident stem cell niches could be identified in the skin biopsies of diffuse cutaneous SSc, indicating that TC loss might be involved in the depletion of functional stem cell compartments in damaged skin, eventually leading to impaired stem cell-mediated skin repair and regeneration in SSc 75. In fact, the loss of TCs is not restricted to the skin, but has also been demonstrated in fibrotic areas of myocardium, lung and gastric wall of SSc patients 74. Further functional studies are required to clarify the roles of TCs in the pathophysiology of SSc. Their potential therapeutic values in stem cell-mediated skin repair and regeneration need to be further investigated.

### Meninges and choroid plexus

Adult neurogenesis is the process of generating new functional neurons from neural stem cells (NSC) and neural progenitor cells (NPC) to react and adapt to extra stimuli in various physiological, pathological and pharmacological conditions 117. NSC and NPC, predominantly located in the subventricular zone of the lateral ventricles and the subgranular zone of the dentate gyrus in the hippocampus, can be activated to proliferate, migrate and differentiate to new neurons 118. However, increasing evidence has indicated that NSC and NPC can also be activated in nonconventional neurogenic zones such as meninges and choroid plexus 119,120. It has previously been proved that TCs are present in foetal and adult rat meninges and choroid plexus 43. Telocytes are more abundant in foetal meninges than in adult meninges, which might be correlated with a higher activity of foetal meningeal stem cells responsible for pre-natal brain development 43. In addition, TCs were found located in the interstitial space between ependymal cells and fenestrated capillaries in adult choroid plexus, in tandem with stem cells by direct cell-to-cell contacts, suggesting the functional roles of TCs in intercellular signalling and neuroregeneration 43. However, to date these roles of TCs have merely been suggested based on their morphological features and ‘strategic’ distribution in meninges and choroid plexus. Thus, in future studies it is highly needed to further investigate and prove the potential involvement of TCs in the intercellular communication as well as in the proliferation, migration or differentiation of stem cells during neurogenesis.

### Eye

Stem cell therapies are considered as innovative approaches for preserving or restoring vision for patients with eye diseases 121. Stem cells and progenitor cells have been found abundant in limbus 122–125, and have also been identified in the ciliary body epithelium, ciliary marginal zone, iris and retina 126–130. Stem cell transplantation, particularly in the cornea, the neural retina or the retinal pigment epithelium, is aimed to either directly replace lost or damaged tissue, or replace essential functions of the tissue 121,131. Despite the encouraging results from basic researches and limited preclinical and Phase I/II trails, cellular mechanisms responsible for eye regeneration and repair are still far from elucidated.

**Figure 9:**
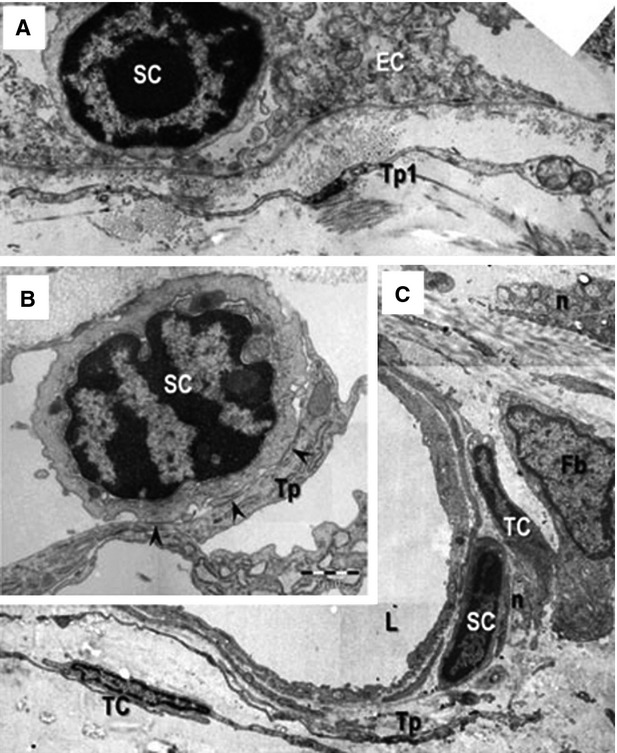
Telocytes bordering stem cells in eye. Telocytes (TCs) bordering stem cells in epithelial (A) and stromal (B and C) stem cell (SC) niches in mouse eye. bl: basal lamina; Tp: Telopode; Fb: fibroblast; n: nerve endings; L: lumen of an arteriole. Scale bars: 2 μm (A); 1 μm (B); 5 μm (C). Reproduced with permission from 56.

Previously, the presence of TCs and stem cells in the limbus and uvea of mouse eye has been demonstrated 56 (Fig.9). Telocytes can form homocellular adhaerens and gap junctions with each other, as well as heterocellular junctions (membrane-to-membrane point contacts or planar contacts) with stem cells, melanocytes, macrophages, fibroblasts, nerve endings and capillaries 56. Telocytes and stem cells co-exist in the stem cell niches located in the limbus and iris stroma, suggesting the possible roles of TCs in eye regeneration and repair. In addition, extracellular vesicles have been found near TCs, indicating that TCs might actively participate in the intercellular communication and signalling among neighbouring cells and within stem cell niche 56. In the future, it is important to gain further knowledge about the exact mechanisms by which TCs might affect the biological functions and fate of stem cells during eye regeneration and repair. Last but not least, given that the potential roles of TCs in neo-angiogenesis after AMI has been demonstrated in heart 100 and that TCs secret VEGF 100 and express PDGFR-β in heart 26, skeletal muscle 19, lungs 107 and liver 57,78, the potential of TCs in neovascular eye diseases deserves to be further investigated 132.

### Liver

Liver possesses an extraordinary capacity to regenerate after toxic or drug-induced injury, virus infection, ischaemia and surgical resection 133,134. The proliferation of remaining hepatocytes and the activation of liver stem/progenitor cells represent two main cellular mechanisms during liver regeneration 135,136. The presence of TCs in liver has previously been demonstrated by transmission electron microscopy and immunofluorescent staining (double labelling for CD34 and c-kit/CD117, or vimentin, or PDGFR-α, or β) 57. Telocytes are located in the Disse space of liver, in close association with hepatocytes, endothelial cells and putative stem/progenitor cells 57.

The potential roles of TCs in liver regeneration have recently been demonstrated by using a murine model of partial hepatectomy (PH) 78. The peak activity of hepatic cell proliferation occurred at 48 hrs post PH (although a remarkable high level of hepatic cell proliferation was also present at 72 hrs post PH). Meanwhile, the number of TCs and CK-19-positive liver stem cells peaked at 72 hrs post PH. These results indicated a close relationship between TCs and the cells essentially implicated in liver regeneration, probably by influencing either the proliferation of hepatocytes or the activation of stem/progenitor cells or even both of them. However, further studies are highly desirable to find the direct evidence for the functional effects of TCs in liver regeneration. Moreover, as TCs have recently been found to be reduced in human liver fibrosis which could be responsible for the aberrant activation of fibroblasts, adding TCs is supposed to be a potential targeted antifibrotic therapy for liver fibrosis 137.

### Uterus

The presence and functions of TCs in human non-pregnant and pregnant myometrium have previously been demonstrated 11,13,51,138. TCs were found involved as modulators in the contractile mechanisms of uterus 11,12,139. Small-conductance calcium-activated potassium (SK3) channels are expressed in human myometrium, which contribute to the relaxation of human myometrium *in vitro* 140. Recently, SK3 channels have been found expressed in CD34-positive TCs in human non-pregnant myometrium 12. However, CD34-positive TCs lacked SK3 channel expression in pregnant myometrium, which indicated that SK3 channel modulation may be involved in myometrial contractility during pregnancy through influencing TCs 12. In addition, T-type calcium channels have been identified in TCs from human myometrium 141. Telocytes have also been shown to be involved in the regulation of foetal blood flow and intra-placental blood volume in physiological or pathological pregnancy (*e.g*. preeclampsia) 142,143. By transmission electron microscopy, ultrastructural evidence of 3D network formed by homocellular and heterocellular contacts of TCs has been demonstrated in human myometrium 13,51. Because of the different physiological state of non-pregnant and pregnant uterus, Tps are thinner and longer in non-pregnant myometrium compared to those in pregnant myometrium 13,51. Shed vesicles and/or exosomes have also been found along Tps or releasing from them in myometrium as described in other tissues 13,51.

In addition, TCs have also been identified in human endometrium tissues, indicating that TCs may support the structure of the stratum functionalis of endometrium 50. Meanwhile, endometrial TCs isolated and cultured from the rat uterus were found connected to nearby or distant stromal cells, suggesting the active roles of TCs in endometrial homeostasis and maintenance through cell-to-cell communication 50.

The formation, elongation, deviation and ramification of Tps may importantly modulate the morphology and the function of TCs, as well as influence their contacts with per se or other cells. Optical stimulation and guidance of neuronal cells which aimed to modify the neuronal growth by using low-level laser stimulation (LLLS) has extensively been studied 144. It has recently been documented that LLLS could also induce a higher growth rate as well as more angled deviation of telopodal lateral extension (TLE) in pregnant myometrium primary cultures when compared to those in non-pregnant myometrium ones, which was supposed to be related to distinct cytoskeleton characteristics of TCs and modified cell-to-cell communication between pregnant and non-pregnant myometrium 52. Since uterine remodelling in pregnancy is associated with morphological and functional changes of multiple cells, such as smooth muscle cells and interstitial cells including TCs, and that TCs actively contribute to form a 3D interstitial network in myometrium, the modulatory effects of LLLS on TLE growth might be a potential intervention or therapeutic strategy in uterine regenerative medicine.

### Urinary system

It has previously been reported that TCs are present in the upper lamina propria (ULP) of the human renal pelvis, ureter and urethra, as well as in kidney and urinary bladder 14,41,42. As ULP layer is located just underneath the urothelium, ULP interstitial cells might be involved in the conduct and amplification of pacemaker signals as well as in the pathophysiology of urinary system disorders 145–147. Telocytes in the ULP of renal pelvis, ureter and urethra have similar ultrastructural features which were different from those of bladder TCs: (*i*) thinner and longer cytoplasmic prolongations; (*ii*) presence of dense core granules and microtubules and (*iii*) no peripheral actin filaments 14. The differences in the ultrastructural phenotype of TCs indicate that each region of the urinary tract might contain its subtype of TCs with probably particular functions 14,148. Interestingly, different expressions of ER and PR have been detected in TCs of the renal pelvis, ureter, bladder and urethra, indicating that the function of ULP TCs might be potentially related to steroid hormones 14. In addition, TCs could establish close contacts with macrophages in sub-capsular space of kidney, and with smooth muscle bundles, blood vessels and nerve endings in ureter and urinary bladder 41. Telocytes have also been identified around renal tubules and vessels in the kidney cortex interstitium, with shed vesicles identified in close vicinity of TCs 42.

The presence of TCs in the urinary system also suggest their potential involvement in the repair and regeneration of injured tissues during diseases such as acute renal failure. Previously, it has been reported that injection of TCs *via* caudal vein was effective to reduce histological renal damage and attenuate renal dysfunction after renal ischaemia-reperfusion injury (IRI) in rats, which might be partially related to the secretion of growth factors other than anti-inflammatory mechanisms 82. However, TCs could not protect renal tubular epithelial cells *in vitro* probably because of their insufficient paracrine ability of growth factors in such circumstance 82. These results suggest that only by supporting and interacting with other cells in microenvironments could TCs exert their effects on the repair and regeneration of renal tubules following renal IRI 82. However, the mechanisms by which TCs contribute to the repair of ischaemically injured renal tubules remain to be further studied.

## Targeting TCs as a potential therapeutic strategy

Telocytes have been found to be decreased in experimental MI, especially in fibrotic areas 101,102. Intramyocardial transplantation of cardiac TCs could decrease MI and improve post-infarcted cardiac function *via* increasing cardiac angiogenesis, improving reconstruction of the TC network and decreasing cardiac fibrosis 101,102. Although the direct effect of cardiac TCs in regulating angiogenesis is unclear, human lung TCs have been reported to promote the proliferation and angiogenesis of human pulmonary microvascular endothelial cells *in vitro* 149. Moreover, as several reports have indicated that TCs were decreased in fibrotic remodelling, such as SSc, liver fibrosis, and ulcerative colitis, it is urgent to determine if TCs could prevent the activation of fibroblasts and attenuate the altered organization of extracellular matrix during fibrotic processes 74,150. Given that endometriosis-affected rat oviduct displayed damaged TCs which might be related to impaired stem cell-mediated tissue repair, we might promote regeneration and prevent the evolution to irreversible tissue damage by targeting TCs alone or in tandem with stem cells 151. Despite that LLLS has been reported to accelerate the growth of TLE in pregnant myometrium primary cultures 52, exploring other pharmacological or non-pharmacological methods to enhance the growth of TCs could be regarded as a novel therapeutic strategy besides exogenous transplantation for disorders.

## Summary

In summary, we have systemically reviewed the most recent studies of the potential significance of TCs in tissue repair and regeneration in heart, lung, skeletal muscle, skin, meninges and choroid plexus, eye, liver, uterus and urinary system. However, it has to be noted that up till now, the studies of TCs were mainly based on the morphology aspect, while direct evidence to show the roles of TCs in diseases and regenerative medicine is still lacking. In such circumstances, it is necessary to explore TC-specific markers useful to identify the presence of TCs in a more specific and easier way in tissues. In addition, it is highly desirable to perform more functional studies, for example the gain-of-function assay *via* transplantation of TCs and the loss-of-function assay *via* specific inhibition of the biological functions of TCs in animal models *in vivo*, as well as the primary culture of TCs from different tissues, the co-culture of TCs with other cells and the use of 3D culture environment and tissue engineering *in vitro*, to further confirm and clarify the mechanisms by which TCs contribute to tissue repair and regeneration. Also, a deeper understanding of the relationship between TCs and stem/progenitor cells is of great need. All these studies will be useful to clarify the biological functions of TCs in tissue repair and regeneration, as well as to provide new insights into the potential therapeutic values of TCs in regenerative medicine.

## References

[b1] Popescu LM, Faussone-Pellegrini MS (2010). TELOCYTES - a case of serendipity: the winding way from Interstitial Cells of Cajal (ICC), *via* Interstitial Cajal-Like Cells (ICLC) to TELOCYTES. J Cell Mol Med.

[b2] Zheng Y, Cretoiu D, Yan G (2014). Protein profiling of human lung telocytes and microvascular endothelial cells using iTRAQ quantitative proteomics. J Cell Mol Med.

[b3] Zheng Y, Cretoiu D, Yan G (2014). Comparative proteomic analysis of human lung telocytes with fibroblasts. J Cell Mol Med.

[b4] Zheng Y, Zhang M, Qian M (2013). Genetic comparison of mouse lung telocytes with mesenchymal stem cells and fibroblasts. J Cell Mol Med.

[b5] Sun X, Zheng M, Zhang M (2014). Differences in the expression of chromosome 1 genes between lung telocytes and other cells: mesenchymal stem cells, fibroblasts, alveolar type II cells, airway epithelial cells and lymphocytes. J Cell Mol Med.

[b6] Zheng M, Sun X, Zhang M (2014). Variations of chromosomes 2 and 3 gene expression profiles among pulmonary telocytes, pneumocytes, airway cells, mesenchymal stem cells and lymphocytes. J Cell Mol Med.

[b7] Cretoiu SM, Popescu LM (2014). Telocytes revisited. Biomol Concepts.

[b8] Ciontea SM, Radu E, Regalia T (2005). C-kit immunopositive interstitial cells (Cajal-type) in human myometrium. J Cell Mol Med.

[b9] Cretoiu D, Hummel E, Zimmermann H (2014). Human cardiac telocytes: 3D imaging by FIB-SEM tomography. J Cell Mol Med.

[b10] Pellegrini M-SF, Popescu LM (2011). Telocytes. Biomol Concepts.

[b11] Cretoiu SM, Simionescu AA, Caravia L (2011). Complex effects of imatinib on spontaneous and oxytocin-induced contractions in human non-pregnant myometrium. Acta Physiol Hung.

[b12] Rosenbaum ST, Svalø J, Nielsen K (2012). Immunolocalization and expression of small-conductance calcium-activated potassium channels in human myometrium. J Cell Mol Med.

[b13] Cretoiu SM, Cretoiu D, Marin A (2013). Telocytes: ultrastructural, immunohistochemical and electrophysiological characteristics in human myometrium. Reprod Camb Engl.

[b14] Gevaert T, De Vos R, Van Der Aa F (2012). Identification of telocytes in the upper lamina propria of the human urinary tract. J Cell Mol Med.

[b15] Popescu LM, Manole CG, Gherghiceanu M (2010). Telocytes in human epicardium. J Cell Mol Med.

[b16] Suciu L, Popescu LM, Gherghiceanu M (2010). Telocytes in human term placenta: morphology and phenotype. Cells Tissues Organs.

[b17] Zhou Y, Pan P, Yao L (2010). CD117-positive cells of the heart: progenitor cells or mast cells?. J Histochem Cytochem.

[b18] Bei Y, Zhou Q, Fu S (2015). Cardiac telocytes and fibroblasts in primary culture: different morphologies and immunophenotypes. PLoS ONE.

[b19] Suciu LC, Popescu BO, Kostin S (2012). Platelet-derived growth factor receptor-β-positive telocytes in skeletal muscle interstitium. J Cell Mol Med.

[b20] Nees S, Weiss DR, Juchem G (2013). Focus on cardiac pericytes. Pflugers Arch.

[b21] Vannucchi M-G, Traini C, Manetti M (2013). Telocytes express PDGFRα in the human gastrointestinal tract. J Cell Mol Med.

[b22] Povýšil C, Kaňa M, Zámečník L (2014). Podoplanin (D2-40) is a reliable marker of urinary bladder myofibroblasts (telocytes). Folia Biol (Praha).

[b23] Kostin S (2010). Myocardial telocytes: a specific new cellular entity. J Cell Mol Med.

[b24] Faussone-Pellegrini MS, Bani D (2010). Relationships between telocytes and cardiomyocytes during pre- and post-natal life. J Cell Mol Med.

[b25] Gherghiceanu M, Manole CG, Popescu LM (2010). Telocytes in endocardium: electron microscope evidence. J Cell Mol Med.

[b26] Yang Y, Sun W, Wu SM (2014). Telocytes in human heart valves. J Cell Mol Med.

[b27] Cantarero I, Luesma MJ, Junquera C (2011). The primary cilium of telocytes in the vasculature: electron microscope imaging. J Cell Mol Med.

[b28] Li H, Lu S, Liu H (2014). Scanning electron microscope evidence of telocytes in vasculature. J Cell Mol Med.

[b29] Zhang HQ, Lu SS, Xu T (2015). Morphological evidence of telocytes in mice aorta. Chin Med J (Engl.).

[b30] Hinescu ME, Gherghiceanu M, Suciu L (2011). Telocytes in pleura: two- and three-dimensional imaging by transmission electron microscopy. Cell Tissue Res.

[b31] Zheng Y, Li H, Manole CG (2011). Telocytes in trachea and lungs. J Cell Mol Med.

[b32] Popescu LM, Gherghiceanu M, Suciu LC (2011). Telocytes and putative stem cells in the lungs: electron microscopy, electron tomography and laser scanning microscopy. Cell Tissue Res.

[b33] Rusu MC, Jianu AM, Mirancea N (2012). Tracheal telocytes. J Cell Mol Med.

[b34] Popescu LM, Manole E, Serboiu CS (2011). Identification of telocytes in skeletal muscle interstitium: implication for muscle regeneration. J Cell Mol Med.

[b35] Bojin FM, Gavriliuc OI, Cristea MI (2011). Telocytes within human skeletal muscle stem cell niche. J Cell Mol Med.

[b36] Chen X, Zheng Y, Manole CG (2013). Telocytes in human oesophagus. J Cell Mol Med.

[b37] Rusu MC, Nicolescu MI, Jianu AM (2012). Esophageal telocytes and hybrid morphologies. Cell Biol Int.

[b38] Cantarero Carmona I, Luesma Bartolomé MJ, Junquera Escribano C (2011). Identification of telocytes in the lamina propria of rat duodenum: transmission electron microscopy. J Cell Mol Med.

[b39] Cretoiu D, Cretoiu SM, Simionescu AA (2012). Telocytes, a distinct type of cell among the stromal cells present in the lamina propria of jejunum. Histol Histopathol.

[b40] Díaz-Flores L, Gutiérrez R, García MP (2015). Human resident CD34^+^ stromal cells/telocytes have progenitor capacity and are a source of αsma+ cells during repair. Histol Histopathol.

[b41] Zheng Y, Zhu T, Lin M (2012). Telocytes in the urinary system. J Transl Med.

[b42] Qi G, Lin M, Xu M (2012). Telocytes in the human kidney cortex. J Cell Mol Med.

[b43] Popescu BO, Gherghiceanu M, Kostin S (2012). Telocytes in meninges and choroid plexus. Neurosci Lett.

[b44] Díaz-Flores L, Gutiérrez R, Sáez FJ (2013). Telocytes in neuromuscular spindles. J Cell Mol Med.

[b45] Rusu MC, Pop F, Hostiuc S (2011). The human trigeminal ganglion: c-kit positive neurons and interstitial cells. Ann Anat.

[b46] Nicolescu MI, Popescu LM (2012). Telocytes in the interstitium of human exocrine pancreas: ultrastructural evidence. Pancreas.

[b47] Bosco C, Díaz E, Gutiérrez R (2013). Ganglionar nervous cells and telocytes in the pancreas of Octodon degus: extra and intrapancreatic ganglionar cells and telocytes in the degus. Auton Neurosci Basic Clin.

[b48] Nicolescu MI, Bucur A, Dinca O (2012). Telocytes in parotid glands. Anat Rec (Hoboken).

[b49] Corradi LS, Jesus MM, Fochi RA (2013). Structural and ultrastructural evidence for telocytes in prostate stroma. J Cell Mol Med.

[b50] Hatta K, Huang ML, Weisel RD (2012). Culture of rat endometrial telocytes. J Cell Mol Med.

[b51] Creţoiu SM, Creţoiu D, Popescu LM (2012). Human myometrium - the ultrastructural 3D network of telocytes. J Cell Mol Med.

[b52] Campeanu RA, Radu BM, Cretoiu SM (2014). Near-infrared low-level laser stimulation of telocytes from human myometrium. Lasers Med Sci.

[b53] Ullah S, Yang P, Zhang L (2014). Identification and characterization of telocytes in the uterus of the oviduct in the Chinese soft-shelled turtle, Pelodiscus sinensis: TEM evidence. J Cell Mol Med.

[b54] Ceafalan L, Gherghiceanu M, Popescu LM (2012). Telocytes in human skin–are they involved in skin regeneration?. J Cell Mol Med.

[b55] Cretoiu D, Gherghiceanu M, Hummel E (2015). FIB-SEM tomography of human skin telocytes and their extracellular vesicles. J Cell Mol Med.

[b56] Luesma MJ, Gherghiceanu M, Popescu LM (2013). Telocytes and stem cells in limbus and uvea of mouse eye. J Cell Mol Med.

[b57] Xiao J, Wang F, Liu Z (2013). Telocytes in liver: electron microscopic and immunofluorescent evidence. J Cell Mol Med.

[b58] Li H, Zhang H, Yang L (2014). Telocytes in mice bone marrow: electron microscope evidence. J Cell Mol Med.

[b59] Gherghiceanu M, Popescu LM (2012). Cardiac telocytes - their junctions and functional implications. Cell Tissue Res.

[b60] Zhou J, Zhang Y, Wen X (2010). Telocytes accompanying cardiomyocyte in primary culture: two- and three-dimensional culture environment. J Cell Mol Med.

[b61] Fertig ET, Gherghiceanu M, Popescu LM (2014). Extracellular vesicles release by cardiac telocytes: electron microscopy and electron tomography. J Cell Mol Med.

[b62] Cismasiu VB, Popescu LM (2015). Telocytes transfer extracellular vesicles loaded with microRNAs to stem cells. J Cell Mol Med.

[b63] Díaz-Flores L, Gutiérrez R, García MP (2014). Uptake and intracytoplasmic storage of pigmented particles by human CD34^+^ stromal cells/telocytes: endocytic property of telocytes. J Cell Mol Med.

[b64] Bani D, Formigli L, Gherghiceanu M (2010). Telocytes as supporting cells for myocardial tissue organization in developing and adult heart. J Cell Mol Med.

[b65] Edelstein L, Smythies J (2014). The role of telocytes in morphogenetic bioelectrical signaling: once more unto the breach. Front Mol Neurosci.

[b66] Mandache E, Gherghiceanu M, Macarie C (2010). Telocytes in human isolated atrial amyloidosis: ultrastructural remodelling. J Cell Mol Med.

[b67] Zheng Y, Bai C, Wang X (2012). Telocyte morphologies and potential roles in diseases. J Cell Physiol.

[b68] Ardeleanu C, Bussolati G (2011). Telocytes are the common cell of origin of both PEComas and GISTs: an evidence-supported hypothesis. J Cell Mol Med.

[b69] Mou Y, Wang Y, Li J (2013). Immunohistochemical characterization and functional identification of mammary gland telocytes in the self-assembly of reconstituted breast cancer tissue *in vitro*. J Cell Mol Med.

[b70] Zheng Y, Bai C, Wang X (2012). Potential significance of telocytes in the pathogenesis of lung diseases. Expert Rev Respir Med.

[b71] Matyja A, Gil K, Pasternak A (2013). Telocytes: new insight into the pathogenesis of gallstone disease. J Cell Mol Med.

[b72] Milia AF, Ruffo M, Manetti M (2013). Telocytes in Crohn’s disease. J Cell Mol Med.

[b73] Mirancea N, Moroşanu A-M, Mirancea G-V (2013). Infrastructure of the telocytes from tumor stroma in the skin basal and squamous cell carcinomas. Romanian J Morphol Embryol.

[b74] Manetti M, Rosa I, Messerini L (2014). A loss of telocytes accompanies fibrosis of multiple organs in systemic sclerosis. J Cell Mol Med.

[b75] Manetti M, Guiducci S, Ruffo M (2013). Evidence for progressive reduction and loss of telocytes in the dermal cellular network of systemic sclerosis. J Cell Mol Med.

[b76] Gherghiceanu M, Popescu LM (2010). Cardiomyocyte precursors and telocytes in epicardial stem cell niche: electron microscope images. J Cell Mol Med.

[b77] Popescu LM, Nicolescu MI, Goldenberg RCS, de Carvalho ACC (2013). Chapter 11 - telocytes and stem cells. Resident stem cells regenerative therapy.

[b78] Wang F, Song Y, Bei Y (2014). Telocytes in liver regeneration: possible roles. J Cell Mol Med.

[b79] Matsa E, Burridge PW, Wu JC (2014). Human stem cells for modeling heart disease and for drug discovery. Sci Transl Med.

[b80] Zhang H, Chen H, Wang W (2010). Cell survival and redistribution after transplantation into damaged myocardium. J Cell Mol Med.

[b81] Drummond-Barbosa D (2008). Stem cells, their niches and the systemic environment: an aging network. Genetics.

[b82] Li L, Lin M, Li L (2014). Renal telocytes contribute to the repair of ischemically injured renal tubules. J Cell Mol Med.

[b83] Segers VFM, Lee RT (2008). Stem-cell therapy for cardiac disease. Nature.

[b84] Ptaszek LM, Mansour M, Ruskin JN (2012). Towards regenerative therapy for cardiac disease. Lancet.

[b85] Christoffels VM, Pu WT (2013). Developing insights into cardiac regeneration. Dev Camb Engl.

[b86] Koudstaal S, Jansen Of Lorkeers SJ, Gaetani R (2013). Concise review: heart regeneration and the role of cardiac stem cells. Stem Cells Transl Med.

[b87] Rosenzweig A (2012). Medicine. Cardiac regeneration. Science.

[b88] Preda MB, Valen G (2013). Evaluation of gene and cell-based therapies for cardiac regeneration. Curr Stem Cell Res Ther.

[b89] Popescu LM, Gherghiceanu M, Manole CG (2009). Cardiac renewing: interstitial Cajal-like cells nurse cardiomyocyte progenitors in epicardial stem cell niches. J Cell Mol Med.

[b90] Popescu LM, Curici A, Wang E (2015). Telocytes and putative stem cells in ageing human heart. J Cell Mol Med.

[b91] Bollini S, Smart N, Riley PR (2011). Resident cardiac progenitor cells: at the heart of regeneration. J Mol Cell Cardiol.

[b92] Bollini S, Vieira JMN, Howard S (2014). Re-activated adult epicardial progenitor cells are a heterogeneous population molecularly distinct from their embryonic counterparts. Stem Cells Dev.

[b93] Niculite CM, Regalia TM, Gherghiceanu M (2015). Dynamics of telopodes (telocyte prolongations) in cell culture depends on extracellular matrix protein. Mol Cell Biochem.

[b94] Hunt GC, Singh P, Schwarzbauer JE (2012). Endogenous production of fibronectin is required for self-renewal of cultured mouse embryonic stem cells. Exp Cell Res.

[b95] Stoffels JMJ, Zhao C, Baron W (2013). Fibronectin in tissue regeneration: timely disassembly of the scaffold is necessary to complete the build. Cell Mol Life Sci.

[b96] Wang J, Karra R, Dickson AL (2013). Fibronectin is deposited by injury-activated epicardial cells and is necessary for zebrafish heart regeneration. Dev Biol.

[b97] Gherghiceanu M, Popescu LM (2011). Heterocellular communication in the heart: electron tomography of telocyte-myocyte junctions. J Cell Mol Med.

[b98] Chugh AR, Beache GM, Loughran JH (2012). Administration of cardiac stem cells in patients with ischemic cardiomyopathy: the SCIPIO trial surgical aspects and interim analysis of myocardial function and viability by magnetic resonance. Circulation.

[b99] Bolli R, Chugh AR, D’Amario D (2011). Cardiac stem cells in patients with ischaemic cardiomyopathy (SCIPIO): initial results of a randomised phase 1 trial. Lancet.

[b100] Manole CG, Cismaşiu V, Gherghiceanu M (2011). Experimental acute myocardial infarction: telocytes involvement in neo-angiogenesis. J Cell Mol Med.

[b101] Zhao B, Chen S, Liu J (2013). Cardiac telocytes were decreased during myocardial infarction and their therapeutic effects for ischaemic heart in rat. J Cell Mol Med.

[b102] Zhao B, Liao Z, Chen S (2014). Intramyocardial transplantation of cardiac telocytes decreases myocardial infarction and improves post-infarcted cardiac function in rats. J Cell Mol Med.

[b103] Miao Q, Shim W, Tee N (2014). iPSC-derived human mesenchymal stem cells improve myocardial strain of infarcted myocardium. J Cell Mol Med.

[b104] Horch RE, Kneser U, Polykandriotis E (2012). Tissue engineering and regenerative medicine -where do we stand?. J Cell Mol Med.

[b105] Zhou J, Wang Y, Zhu P (2014). Distribution and characteristics of telocytes as nurse cells in the architectural organization of engineered heart tissues. Sci China Life Sci.

[b106] Tao L, Wang H, Wang X (2015). Cardiac telocytes. Curr Stem Cell Res Ther.

[b107] Galiger C, Kostin S, Golec A (2014). Phenotypical and ultrastructural features of Oct4-positive cells in the adult mouse lung. J Cell Mol Med.

[b108] Kuang S, Gillespie MA, Rudnicki MA (2008). Niche regulation of muscle satellite cell self-renewal and differentiation. Cell Stem Cell.

[b109] Mitchell KJ, Pannérec A, Cadot B (2010). Identification and characterization of a non-satellite cell muscle resident progenitor during postnatal development. Nat Cell Biol.

[b110] LaBarge MA, Blau HM (2002). Biological progression from adult bone marrow to mononucleate muscle stem cell to multinucleate muscle fiber in response to injury. Cell.

[b111] Doyonnas R, LaBarge MA, Sacco A (2004). Hematopoietic contribution to skeletal muscle regeneration by myelomonocytic precursors. Proc Natl Acad Sci USA.

[b112] Dellavalle A, Sampaolesi M, Tonlorenzi R (2007). Pericytes of human skeletal muscle are myogenic precursors distinct from satellite cells. Nat Cell Biol.

[b113] Rusu MC, Mirancea N, Mănoiu VS (2012). Skin telocytes. Ann Anat.

[b114] Wong VW, Levi B, Rajadas J (2012). Stem cell niches for skin regeneration. Int J Biomater.

[b115] Fuchs E (2008). Skin stem cells: rising to the surface. J Cell Biol.

[b116] Nihtyanova SI, Ong VH, Denton CP (2014). Current management strategies for systemic sclerosis. Clin Exp Rheumatol.

[b117] Ming GL, Song H (2011). Adult neurogenesis in the mammalian brain: significant answers and significant questions. Neuron.

[b118] Zhao C, Deng W, Gage FH (2008). Mechanisms and functional implications of adult neurogenesis. Cell.

[b119] Nakagomi T, Molnár Z, Nakano-Doi A (2011). Ischemia-induced neural stem/progenitor cells in the pia mater following cortical infarction. Stem Cells Dev.

[b120] Itokazu Y, Kitada M, Dezawa M (2006). Choroid plexus ependymal cells host neural progenitor cells in the rat. Glia.

[b121] Blenkinsop TA, Corneo B, Temple S (2012). Ophthalmologic stem cell transplantation therapies. Regen Med.

[b122] Ordonez P, Di Girolamo N (2012). Limbal epithelial stem cells: role of the niche microenvironment. Stem Cells.

[b123] Shortt AJ, Secker GA, Munro PM (2007). Characterization of the limbal epithelial stem cell niche: novel imaging techniques permit *in vivo* observation and targeted biopsy of limbal epithelial stem cells. Stem Cells.

[b124] Pinnamaneni N, Funderburgh JL (2012). Concise review: stem cells in the corneal stroma. Stem Cells.

[b125] Meller D, Thomasen H, Steuhl K-P (2012). Ocular surface reconstruction in limbal stem cell deficiency: transplantation of cultivated limbal epithelium. Ophthalmologe.

[b126] Tropepe V, Coles BLK, Chiasson BJ (2000). Retinal stem cells in the adult mammalian eye. Science.

[b127] Haruta M, Kosaka M, Kanegae Y (2001). Induction of photoreceptor-specific phenotypes in adult mammalian iris tissue. Nat Neurosci.

[b128] Bhatia B, Singhal S, Jayaram H (2010). Adult retinal stem cells revisited. Open Ophthalmol J.

[b129] Du Y, Roh DS, Mann MM (2012). Multipotent stem cells from trabecular meshwork become phagocytic TM cells. Invest Ophthalmol Vis Sci.

[b130] Wohl SG, Schmeer CW, Isenmann S (2012). Neurogenic potential of stem/progenitor-like cells in the adult mammalian eye. Prog Retin Eye Res.

[b131] Ahmad S, Kolli S, Lako M (2010). Stem cell therapies for ocular surface disease. Drug Discov Today.

[b132] Campochiaro PA (2013). Ocular neovascularization. J Mol Med Berl Ger.

[b133] Chistiakov DA (2012). Liver regenerative medicine: advances and challenges. Cells Tissues Organs.

[b134] Duncan AW, Soto-Gutierrez A (2013). Liver repopulation and regeneration: new approaches to old questions. Curr Opin Organ Transplant.

[b135] Kandilis AN, Koskinas J, Tiniakos DG (2010). Liver regeneration: focus on cell types and topographic differences. Eur Surg Res.

[b136] Miyajima A, Tanaka M, Itoh T (2014). Stem/progenitor cells in liver development, homeostasis, regeneration, and reprogramming. Cell Stem Cell.

[b137] Fu S, Wang F, Cao Y (2015). Telocytes in human liver fibrosis. J Cell Mol Med.

[b138] Roatesi I, Radu BM, Cretoiu D (2015). Uterine telocytes: a review of current knowledge. Biol Reprod.

[b139] Allix S, Reyes-Gomez E, Aubin-Houzelstein G (2008). Uterine contractions depend on KIT-positive interstitial cells in the mouse: genetic and pharmacological evidence. Biol Reprod.

[b140] Rosenbaum ST, Larsen T, Joergensen JC (2012). Relaxant effect of a novel calcium-activated potassium channel modulator on human myometrial spontaneous contractility *in vitro*. Acta Physiol Oxf Engl.

[b141] Cretoiu SM, Radu BM, Banciu A (2015). Isolated human uterine telocytes: immunocytochemistry and electrophysiology of T-type calcium channels. Histochem Cell Biol.

[b142] Bosco C, Díaz E, Gutiérrez R (2015). A putative role for telocytes in placental barrier impairment during preeclampsia. Med Hypotheses.

[b143] Bosco C, Díaz E, Gutiérrez R (2015). Placental hypoxia developed during preeclampsia induces telocytes apoptosis in chorionic villi affecting the maternal-fetus metabolic exchange. Curr Stem Cell Res Ther.

[b144] Ebbesen CL, Bruus H (2012). Analysis of laser-induced heating in optical neuronal guidance. J Neurosci Methods.

[b145] Gevaert T, De Vos R, Everaerts W (2011). Characterization of upper lamina propria interstitial cells in bladders from patients with neurogenic detrusor overactivity and bladder pain syndrome. J Cell Mol Med.

[b146] Lang RJ, Klemm MF (2005). Interstitial cell of Cajal-like cells in the upper urinary tract. J Cell Mol Med.

[b147] Sergeant GP, Thornbury KD, McHale NG (2006). Interstitial cells of Cajal in the urethra. J Cell Mol Med.

[b148] Vannucchi M-G, Traini C, Guasti D (2014). Telocytes subtypes in human urinary bladder. J Cell Mol Med.

[b149] Zheng Y, Chen X, Qian M (2014). Human lung telocytes could promote the proliferation and angiogenesis of human pulmonary microvascular endothelial cells *in vitro*. Mol Cell Ther.

[b150] Manetti M, Rosa I, Messerini L (2015). Telocytes are reduced during fibrotic remodelling of the colonic wall in ulcerative colitis. J Cell Mol Med.

[b151] Yang XJ, Yang J, Liu Z (2015). Telocytes damage in endometriosis-affected rat oviduct and potential impact on fertility. J Cell Mol Med.

